# Validation Parameters of the Magnetic Stirrer Method for Pooled Sample Digestion for *Trichinella* spp. in Horse Meat Based on Proficiency Tests Results

**DOI:** 10.3390/ijerph192114356

**Published:** 2022-11-02

**Authors:** Mirosław Różycki, Weronika Korpysa-Dzirba, Aneta Bełcik, Ewa Bilska-Zając, Aneta Gontarczyk, Maciej Kochanowski, Małgorzata Samorek-Pieróg, Jacek Karamon, Selene Rubiola, Francesco Chiesa, Tomasz Cencek

**Affiliations:** 1 Department of Preclinical Sciences and Infectious Diseases, Poznań University of Life Science, ul. Wolynska 33, 60-637 Poznan, Poland; 2 Department of Parasitology and Invasive Diseases, National Veterinary Research Institute in Pulawy, Partyzantow Avenue 57, 24-100 Pulawy, Poland; 3 Department of Veterinary Sciences, University of Turin, Largo Paolo Braccini 2, 10095 Turin, Italy

**Keywords:** horse meat, proficiency tests, *Trichinella* spp., validation

## Abstract

Meat of horses may be infested with nematodes of the genus *Trichinella* spp. and can cause serious disease in humans. Rules for the carcasses sampling of species susceptible to *Trichinella* spp. infection and examination are laid down in Commission Regulation 1375/2015, where the magnetic stirrer method for pooled-sample digestion is recommended (Commission Regulation 1478/2020). All personnel involved in the examination should be properly trained and participate in quality control programs. Proficiency tests (PTs) play a key role in the quality verification process. This paper presents the results of PTs organized for 68 Polish laboratories in 2014–2019. Results were assessed qualitatively at three levels of sample contamination (0, 3, 5 larvae) and quantitatively at one level (5 larvae). The laboratories have achieved the average correct qualitative results 100%, 96.2% and 96.8% for the samples contaminated with 0, 3 and 5 larvae, respectively. In the quantitative evaluation, an average 94.1% of the reported results were correct. The data from PTs enabled us to define, for the first time, validation parameters of the digestion method for the horse meat matrix in a large-scale experiment including: specificity (100%), sensitivity (95.6%), accuracy (97.1%), the limit of detection (LOD) (1.14 ≈ 1) and the limit of quantification (LOQ) (3.42 ≈ 3).

## 1. Introduction

Trichinellosis is one of the major parasitic zoonoses caused by the nematodes of the genus *Trichinella* spp., characterized by a complex epidemiology due to the circulation of the parasite in various environments. There are different reservoirs, sources, and transmission paths of the invasion. For humans, the source of the parasite is raw or undercooked meat containing living larvae. The consumption of unexamined or poorly examined meat may lead to infection in humans. Proper control is a key element in *Trichinella* spp. prevention. This could be achieved by the elimination of infected animals from the food chain or inactivation of the parasite in meat [1]. The main efforts are focused on controlling *Trichinella* spp. in wild boars (*Sus scrofa*), which are recognized as the main source of the disease; however, the meat of common herbivores such as horses, moose, or even beavers may also pose a real threat to public health [2,3].

In recent decades, the consumption of red meat in developed countries has tended to remain at a constant or decreasing level. People reduce their consumption of this type of meat because of its high content of saturated fatty acids and cholesterol. Among food animals, interest in horse meat has slightly increased [4]. In contrast to the most-often consumed red meats, such as beef, veal, or pork, horse meat has low fat and high unsaturated fatty acids’ content [5]. The horse meat industry shares only 0.4% of the global meat market, with an average consumption of 0.1 kg per capita [6]. However, this type of meat is popular in Western Europe, especially in Italy and Belgium, where the level of consumption of horse meat reaches up to 0.88 kg and 0.5 kg per capita per year, respectively [7]. Simultaneously, in many countries, including Poland, there is no tradition of consuming horse meat, and horses are considered a symbol of high social status: they are used for sports, recreation, agriculture, and agritourism, as they generate positive emotions. At present, in Poland, there are only eight horse slaughterhouses, while there are over 70 times the number of pig slaughterhouses. The Polish meat industry does not have adequate facilities to slaughter horses and process this type of meat, and 80–95% of its production is exported [6,7,8]. Poland is a leader in horse meat exports to the European Union, with its main trade partner being Italy, followed by France, Belgium, Austria, and Germany [9]. Safety and quality criteria are essential to ensure that horse meat is fit for human consumption [10,11].

Although, in the 19th century, there were several reports on experimental and natural infections with *Trichinella* spp. larvae in horses, the potential role of horses in the transmission of this parasite was underestimated until 1975, when an outbreak of trichinellosis occurred in Italy and France after eating horse meat [12,13]. As a result of these cases, the artificial digestion method was adopted in the European Union for *Trichinella* spp. detection in horse meat [12]. Outbreaks of human trichinellosis after the consumption of horse meat occasionally occurred in France and Italy and were related to imported horse meat [12,14,15]. Two outbreaks of trichinellosis involving 1073 cases, which occurred in 1985 in France, were most probably related to carcasses imported from slaughterhouses in the United States and West Germany. In 1986 and 1990, trichinellosis outbreaks occurred in Italy, with 300 and 500 cases, respectively; as for the first outbreak, it was suspected that the horse meat was infected by *Trichinella* spp. originating from Yugoslavia or Poland [16]. In 1991, 1993, and 1994, outbreaks related to the consumption of horse meat imported from the US, Canada, and Belgium, respectively, occurred in France [17,18,19]. In 1996, *Trichinella* spp. larvae were found in horse meat exported from Romania to Italy during re-inspection [15]. Also, in 1998, there were two outbreaks of trichinellosis: one in Italy related to the consumption of horse meat imported from Poland and the second one in France, due to horse meat from Yugoslavia [16,20]. There is no simple explanation for the routes of *Trichinella* spp. transmission to horses. It may occur incidentally by grazing in pastures contaminated with infected small animals and rodent carcasses, or on hay containing pieces of rodents, or even through being fed with animal products [21,22]. However, it was empirically proved that moist feces of rats fed with *Trichinella* spp. infested meat may contain larvae that are able to infect other animals [23]. Traceback studies of the Polish veterinary official data indicate two *Trichinella* spp. infections in horses in the last 30 years (in 1998 and 2002). The first case of trichinellosis was detected in Italy and was caused by *T. britovi,* while, in the second one, mixed infection of *T. spiralis* and *T. britovi* was identified in Poland in meat exported to Italy [24]. Trichinellosis outbreaks have an important impact on public health and trade. Those related to horse meat result in collapsing sales of this type of meat after each outbreak. Taking into account all EU countries, over 70% of horse meat is consumed in France and Italy, predominantly raw. The people who eat raw horse meat regularly may do so because the practice is traditionally thought to reinforce health [25]. This explains why outbreaks of horse-related trichinellosis have only occurred in these countries [12]. Control for *Trichinella* spp. larvae in local and imported horses was not mandatory in the EU until 1991, when Directive 91/497/EEC was established, specifying that 1 g of muscle tissue should be tested in the same way as the procedure used for pig meat examination [26]. However, in 1994, the minimum weight of horse meat samples was increased to 5 g [27]. Meat testing for the presence of *Trichinella* spp. using the pooled sample digestion method supported by a magnetic stirrer (MSD) was introduced in the 1996 in EU by Directive 77/96 [27]. In Poland, this method was only used in large establishments exporting to European markets. The principle of the method is relatively simple and consists of releasing the muscle larvae in the process of digestion with artificial gastric juice and subsequent sedimentations. Larvae are identified in sediments under a stereomicroscope with 40–80x zoom. However, this method is heavily dependent on the pepsin quality and the skills of the personnel [28]. The equipment used for meat testing is also of importance. High-quality of *Trichinella* spp. veterinary control is essential to ensure the safety of all types of meat, including horse meat. This can be achieved by well-trained laboratory staff, able to perform examinations effectively and conduct regular equipment maintenance, especially in relation to preventative maintenance, and also by the quality management system introduced in the laboratory, if possible, accreditation according to: ISO 17025:2018; 18743:2015 Standards and regular attendance in external proficiency test (PT) programs [29,30]. Polish National Reference Laboratory (NRL) for trichinellosis provides staff training, reagent approvals, and the transfer of knowledge from the European Union Reference Laboratory for Parasites (EURLP), as well as organising PT for laboratories performing analyses of pig and horse meat for *Trichinella* spp. This article presents the results of PTs performed for the laboratories analysing horse meat for *Trichinella* spp. during a six year period (2014–2019) and assesses validation parameters of the MSD method for the horse meat matrix.

## 2. Materials and Methods

### 2.1. Laboratories

The main task for laboratories analysing horse meat for the presence of *Trichinella* spp. was the meat examination, of which the majority is intended for export. For this reason, all those laboratories were intensively controlled by the importers. Those laboratories were well-equipped and achieved accreditation according to ISO 17025:2018 Standard given by the Polish Centre for Accreditation (PCA) quite early compared to other laboratories performing meat inspection. The competence of the laboratories was confirmed by the examination of sample sets delivered by NRL each year since 2005. Standard Operation Procedures (SOPs) were provided by Regional Veterinary Laboratories (RVLs), with close cooperation with NRL. Equipment was provided by the Local Veterinary Officer (LVO). Laboratories only used reagents approved by the NRL for the examination, and the list of approved suppliers is available on the General Veterinary Inspectorate (GVI) website [31].

At the moment, in Poland there are 12 registered plants authorized to slaughter the *Equidae*. Four of them have suspended their activities in this area. Out of the remaining eight, in two meat processing plants, dealing with horse meat is marginal. The majority of horses in Poland are slaughtered in three large abattoirs in (1) Małopolska and (2) in Greater Poland voivodeship. The detailed data are available on the GVI website [32].

### 2.2. Proficiency Testing

The preparation of PTs for laboratories examining horse meat is the same as that in the case of pork meat sample preparations, but in this case horse meat was used as a matrix. The principles of proficiency testing, including preparing samples and gelatin capsules, the *Trichinella* spp. detection methodology, as well as interpretation of the results, has been described previously regarding the use of pork meat as the matrix [33]. The laboratories participating in the PTs received sets of samples consisting of one negative sample and samples with one, three, and five larvae of *Trichinella spiralis*. The results obtained from examination of samples spiked with one larva were only used to assess the limit of detection (LOD), limit of quantification (LOQ) and overall quality system performance of all laboratories and they were not considered in the laboratories evaluation.

### 2.3. Data Analysis

The data used for analysis were the PT results collected in 2014–2019 from laboratories that participated in PTs (68 in total). Within 5 years of study, the NRL prepared and distributed 217 PT samples (four samples per set including three samples spiked with 1, 3 and 5 larvae and one negative). Samples spiked with 0, 3 and 5 larvae were used for laboratory evaluation (204 samples). Samples spiked with one larvae were used only to assess the overall performance of the quality system in the laboratories in Poland. The reported results were assessed qualitatively and quantitatively.

Qualitative assessment: the results were assessed as conforming if *Trichinella* spp. larvae were found in positive samples but were not detected in samples without larvae; the results were assessed as unacceptable when laboratories failed to detect *Trichinella* spp. larvae in spiked samples or detected larvae in negative samples. Quantitative assessment was established by the European Union Reference Laboratory (EURL) according to ICT (International Commission on Trichinellosis ) recommendations and is based on the absolute difference |Δ| value between the reported results and the reference value [34,35]. The criteria for quantitative result evaluation were as follows: for samples spiked with 3 larvae, the detection of at least 1 larva is considered acceptable; for samples spiked with 5 larvae evaluation criteria were, satisfactory if |Δ| ≤ 2, doubtful if |Δ| = 3, and unsatisfactory if |Δ| > 3. PT results (all levels) were statistically described, including mean value, standard error, median, mode, standard deviation, sample variance, kurtosis, skewness, range, minimum, maximum, sum, and confidence level (95.0%). Obtained data were used to describe the parameters of the MSD used for horse meat analysis. Parameters describing the method were as follows:

Precision is the closeness of agreement between results obtained under specified conditions. The measure of precision is the coefficient of variation (CV) defined by the formula:

(1) $$\mathrm{CV}=\frac{s}{\bar{x}}\;\times \;100\%$$

where s-standard deviation, x _mean_ ≠ 0.

Reproducibility is described as precision under reproducibility conditions and is counted according to the formula: (reproduced results)/(total number of samples) × 100%.

The limit of detection (LOD) is the lowest amount of an analyte to be examined in a matrix that can be detected. LOD for the MSD was established empirically as 1 larva per sample and was confirmed using formula LOD = 3s where “s” denotes the standard deviation. Result given in integers is LOD = 1 confirmed in laboratory practice [10,36].

The limit of quantification (LOQ) is the lowest amount of an analyte to be examined in a matrix that can be quantitatively determined is given as LOQ = 3LOD. In case of classic parasitology, the number of parasitic elements is usually given in integers and, for this reason, the final LOD and LOQ estimation was given in the same manner.

Overall system performance was based on the results of examination of samples contaminated with one larva. These results were not used for evaluation of each laboratory. In this case, the main criterion was the assessment of the percentage of laboratories that correctly assessed the tested sample. The level higher or equal to 75% indicates a properly implemented management system in laboratories and the good quality of their work. Lower values are a signal that there is a need to intensify work related to the quality system in all laboratories in the region.

Sensitivity (SE), specificity (SP) and accuracy (AC) were calculated according to the EN ISO 16140 Standard, using the formulas:

(2) $$\mathrm{SE}=\frac{\left(\mathrm{PA}\right)}{\left(\mathrm{PA}\right)+\left(\mathrm{ND}\right)}\times \;100\%$$

(3) $$\mathrm{SP}=\frac{\left(\mathrm{NA}\right)}{\left(\mathrm{NA}\right)+\left(\mathrm{PD}\right)}\;\times 100\%$$

(4) $$\mathrm{AC}=\frac{\left(\mathrm{PA}\right)}{\left(\mathrm{PA}\right)+\left(\mathrm{PD}\right)}\times \;100\%$$

where the number of true positive results is (PA), number of false negatives (ND), and false positives (PD) [37].

The uncertainty of measurement was calculated according to ISO/IEC Guide 98–3:2008-Part 3 [38]. A guide to the expression of uncertainty in measurement is given as (u) = √ [∑ (xi − μ)^2^/(n × (n − 1))], where: PT results (xi), the mean value of PT results (μ) and several results (n).

## 3. Results

### 3.1. Qualitative Assessment

The total number of laboratories participating in the tests in 2014–2019 was 68, of which 14 participated in 2014, 3 in 2015, 13 in both 2016 and 2017, 8 in 2018, and 17 in 2019. The percentage of correctly assessed samples ranged from 94.9% in 2016 and 2017 up to 100% in 2014, 2015, and 2018. Incorrect results were reported in 2016, 2017 and 2019. In 2017, the lab mixed up the sample codes. The results of the examination of samples contaminated with one larva were excluded from analysis (measure of the quality system). The results of the laboratories in these years are presented in Table 1 and Table 2.

In 2014, 2015 and 2018, the laboratories reported 100% positively assessed results for sample evaluation. In 2015 and 2018, the number of participants was low, at three and eight, respectively.

The laboratories correctly assessed uncontaminated samples. Incorrect results were reported six times (in total on the levels of 0, 3 and 5 larvae). Three incorrect results were reported for samples contaminated with three *Trichinella* spp. larvae: two in 2016 and one in 2017. Three samples at the five larvae contamination levels were incorrectly estimated: one in 2017 and two in 2019. In 2017, incorrect results for level three and five were reported by the same laboratory.

### 3.2. Quantitative Assessment

Laboratories’ evaluation depends on a single mistake, 100% of laboratories passed the PT comparisons in 2014, 2015 and 2018. The lowest percentage of laboratories reporting correct results was observed in 2016 when two out of 13 (15.4%, both on level 3) failed the PT. The same number of laboratories failed PT in 2019 (11.8% both at the contamination level 5).

**Figure 1:**
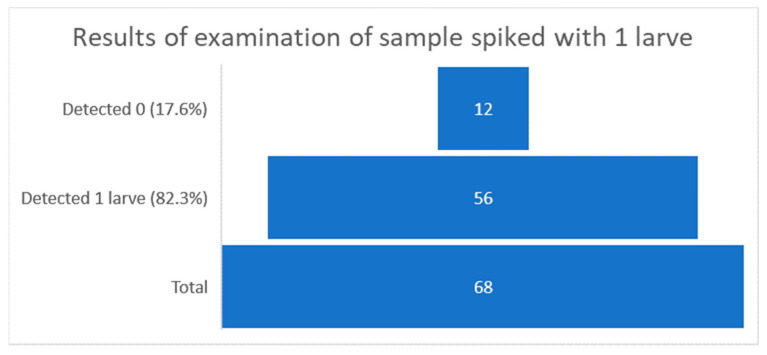
Graphical interpretation of the distribution of sample results examined at the method detection limit (number of laboratories in bricks).

All laboratories reported correct results for uncontaminated samples. PT participants reported 12 incorrect results when examining samples at the method detection limit (samples contaminated with one larva). The distribution of the results at the level of the method detection limit is shown in Figure 1.

**Figure 2:**
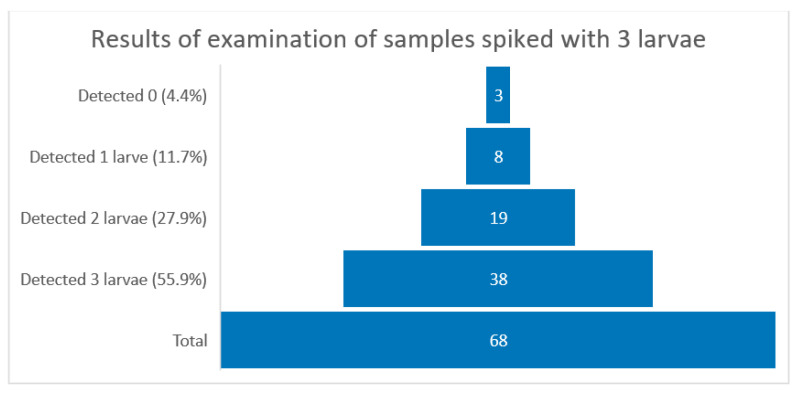
Graphical interpretation of the distribution of samples spiked with three larvae (number of laboratories in bricks).

Over 82% of laboratories (56 out of 68) were able to correctly identify the samples spiked with one larva, which exceeds the assumed minimum and proves a correctly implemented quality system [36]. In case of samples spiked with three larvae, the distribution of reported results was more variable. Three (4.4%) out of 68 laboratories did not find any larvae in the examined samples, eight (11.7%) laboratories reported the presence of one larva, 19 (28%) detected two larvae, and 38 (56%) out of 68 reported exact results. The distribution of the results of testing samples contaminated with three larvae is shown in Figure 2.

**Figure 3:**
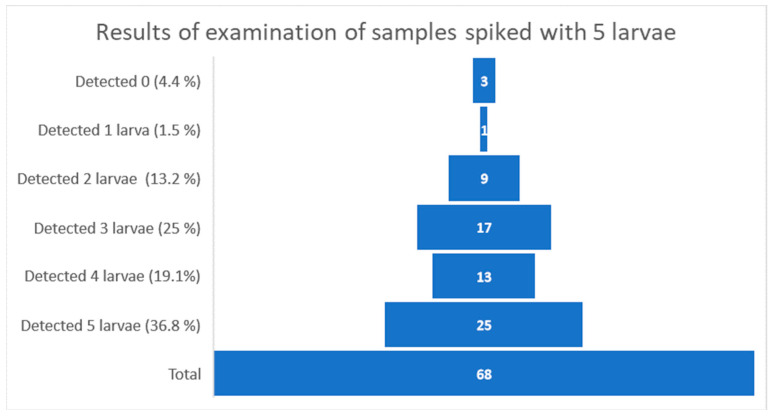
Graphical interpretation of the distribution of samples results spiked with five larvae (number of laboratories in bricks).

Greater diversity in the distribution of results was observed in the group of samples that were contaminated with five larvae, as presented in Figure 3.

Within this group of samples, three laboratories (4.4%) were not able to find a single larva. The detection of one larva was reported once (1.5%), while the detection of two, three, and four *Trichinella* spp. was reported by 9 (13.2%), 17 (25%), and 13 (19.1%) participants, respectively. Correct results were reported for the five detected larvae by 25 (36.8%).

### 3.3. Evaluation of the Reported Results

Evaluation of the reported results was qualitatively and quantitatively performed. Since the suitability of meat for consumption is determined by finding one larva, the method of PT testing is qualitatively assessed. Detailed qualitative results of laboratory evaluation are presented in Table 3.

The reported results were summarized in the form of descriptive statistics based on the real number of detected and reported larvae, as a basic part of a more extensive statistical analysis shown in Table 4.

A quantitative sample assessment was based on the absolute difference |Δ| value between the reported results and the reference value. The results of the quantitative evaluation are presented in Table 5.

For uncontaminated samples, laboratories did not report false-positive results. A sample quantitative assessment revealed the high specificity of the test and confirmed the skills of the personnel and their ability to distinguish larvae from artifacts. Doubtful results considered as positive in the final evaluation are a signal that corrective actions should be undertaken by the laboratory. However, two laboratories reported false–negative results in samples spiked with three larvae, as shown in Table 6. In 2019, one laboratory reported finding one larva in sample contaminated with five larvae. Thus, the laboratory was evaluated positive in qualitative but negative in quantitative assessment.

The double negative results obtained by a single laboratory were reported only once in 2017, when the laboratory missed the sample codes. The results obtained for PTs were used to calculate the precision and coefficient of variation. The results are presented in Table 7. A low CV% indicates the very good precision of laboratory performance and the examined reference samples; CV values lower than 25% indicates very low variability of reported results.

The LOD was established as being equal to 1 (1.14), thus the LOQ as 3LOD was established as 3 (3.42) larvae per sample [36].

The results of the laboratories in relation to the contamination level were used to calculate the sensitivity, specificity, and accuracy of the horse meat MSD. These results are presented in Table 8.

Positive agreement (recovery) PA was confirmed for 130 results. False-positive recovery from samples was PD = 0. Negative agreement (recovery) was NA = 68. False-negative recovery from samples showed ND = 6. The total number of positive results (PA+ND) was 136 and the total number of negative results (NA+PD) was 68. The total number of samples (NA+PA+PD+ND) was 204. These parameters were used to calculate the sensitivity, specificity and accuracy with ULC and LCL, as shown in Table 9.

Data from the proficiency tests provide information that enabled evaluation of uncertainty. Uncertainty was calculated for samples with low and high contamination levels. The equitation results slightly differed, from 0.1 for low samples, to 0.17 for the samples contaminated by *Trichinella* spp. at a high level.

## 4. Discussion

In the case of Poland, one of the leading exporters of horse meat, the quality and safety of such meat is essential to ensure safe meat for humans and therefore a guarantee for international trade. Outbreaks of *Trichinella* spp. linked to the consumption of horse meat may have serious consequences for public health and the horse meat market, as well as in legal and administrative terms, including the implementation of control measures at national and/or international levels. Compared to the number of laboratories that participate in PTs to detect *Trichinella* spp. in pork, as organized by the Polish NRL for trichinellosis, fewer laboratories participate in such PTs if the matrix is horse meat. This difference is related to the situation in the Polish meat market, where pork is consumed frequently, while horse meat is mostly intended for export. Nevertheless, a low number of laboratories analyse horse meat for the presence of *Trichinella* spp. using MSD. The competencies confirmed by participation in PTs are essential when ensuring the safety of horse meat for internal and external markets. The results provide information on the intra-laboratory shortcomings and overall performance of the method. The deficiencies of the MSD were broadly examined. The main sources of are found to be related to: improper sampling, transposition, application of the MSD protocol and equipment [10,39,40].

The very first validation attempts were described in the early 1990s by Prost and Nowakowski, and focused on the equipment as a source of errors [39]. The method was previously broadly studied, and the sensitivity of the method was established by Forbes, Rossi, and Mayer-Sholl for both pork and horse matrices [10,41,42]. However, quantitative data from the PTs in Germany (2008–2010) show that, on average, only 60% of *Trichinella* spp. larvae were detected, and laboratories frequently reported samples with an unexpectedly low larval count (loss of >2 larvae) [43]. General improvements in the quality of the examination have been observed over Europe. The percentage of European NRLs that passed the proficiency test increased from 83% to 100% [34]. General improvements at the national level were also observed and, on average, 80% of positive samples were correctly assessed [40,43,44,45,46,47]. The majority of the available data are for pork; thus, the limited data that are available on PTs for *Trichinella* spp. in horse meat do not allow for comparison of the results, including results focusing only on horse meat. However, these results can be discussed in relation to the MSD used in the PTs to detect *Trichinella* spp. in different types of tested meat. Considering the qualitative evaluation, the high percentage of correctly assessed horse meat samples within the analysed period of time (2014–2019) at the level from 95% to 100% was similar to the results of PT’s performed in pork meat (2015–2019), where this level varied from 94% to 96%. In qualitative evaluation, there were no incorrect results for horse meat. In the case of pork meat, incorrect results were reported by up to 4% of participants. Due to the much lower number of tested horse meat samples, the percentage of incorrect results is quite high in certain years, and may be misleading, especially in 2016 and 2019, when incorrect samples were reported by 15.4% of participants when there were three larvae and 17.6% with five larvae, respectively. However, in these years, the number of laboratories that participated in the PTs was 13 in 2016 and 17 in 2019. A similar situation may be observed in 2017, when only single participants obtained incorrect results in the analysis of horse meat samples contaminated at low (three larvae) and high (five larvae) levels, but because the number of all participants was 13, one incorrect result, when presented as a percentage, gave the total result of 7.7%. The mean value for horse meat samples is higher at both levels, which means that the whole set of collected results represented by a single number is closer to a reference value. The standard error for horse meat is higher compared to pork, which means that the pork sample mean value was closer to the population value but not the reference value. Interestingly, the median value for both types in the matrix was close to the reference value; however, the median for horse meat samples spiked with three larvae was exactly the same as the reference value. In the mode value (most often value in a set) for both types, the matrix was the same as a reference value. The standard deviation value was almost similar for both types of samples; however, for horse meat, the value was slightly lower. Sample variance values for horse meat samples were slightly lower compared to pork samples, due to the smaller spread of PT results around the mean value. The results reported for horse meat at both levels were positive, whereas, in the case of pork, these results were positive and negative. For both matrices, this value did not reach 3; thus, the normality of distribution might be described as mesokurtic with flat outliers. Skewness was negative for both matrices; this type of skewness is called left-tailed distribution, which shows that the mass of the distribution is concentrated on the right side, meaning that laboratories “lose” the larvae more often, instead of finding it. Considering the range values, it can be seen that horse laboratories performed better examinations a than pork ones. These results are presented in Table 10.

The basic measures of performance that are most commonly used for test equitation are accuracy, sensitivity, and specificity. These parameters, when used together, provide distinct and valuable information.

The evaluated results indicate that the performance of laboratories examining horse meat in Poland is better than those controlling pig meat (Table 11). All the validation parameters were higher (specificity by 2.7%, sensitivity by 9.1%, and accuracy by 7.9%). The calculated limits of detection and quantification were almost the same for both matrices.

The obtained results complete the validation results provided by Forbes, focused on system sensitivity and sample size, taken from individual infected carcasses, as well as a description of the critical control points [10]. The data obtained from horses indicate that the currently accepted sample size of 5 g from individual carcasses should provide an acceptable level of safety, since the LOQ is equal to 3.4 g. This observation supports the established five-gram optimum [36]. In addition, validation data support the country’s risk management by identifying and implementing risk mitigation measures to a target, achieving an appropriate level of protection and ensuring the minimization of any negative effects on health and trade. The better results obtained in PTs by laboratories examining horse meat may be explained by the accreditation of all laboratories participating in the trials [48]. The established tradition of examining horse meat for export goes back to the early 1980s: training and participation in PTs and verification programs to confirm their high competencies.

The MSD method is standardized and obligatory for meat inspection in Europe; however, still there is lack of validation data. The presented results complement the gap in the validation common for parasitological methods used for official purposes [49]. These quantitative data can support risk management at the national and international levels.

## 5. Conclusions

The presented results indicate that the quality of analysis performed by the laboratories examining horse meat for the presence of *Trichinella* spp. in Poland is very high. The collected data enabled calculation of the validation parameters in relation to horse meat and are very useful for establishing the acceptance criteria for confirming the validity of the results. The presented data highly support the official quality control program of the test used to detect *Trichinella* spp. in horse meat and are helpful in filling the requirements of the Article 5 1375/2015 Regulation. This publication provides valuable validation data obtained for the first time from field laboratories in the so-called large-scale experiment and according to our knowledge such an evaluation has not been published previously in Europe in relation to the MSD method and horse meat matrix.

## Figures and Tables

**Table 1:** Sample qualitative results of PT from 2014 to 2019.

Year	Number of Laboratories Participating in the Study	Total Number of Sent Samples (Contamination Levels 0, 3, 5)	Total Number of Samples Correctly Assessed	Total Number of Samples Incorrectly Assessed	% of Samples Correctly Assessed
2014	14	42	42	0	100
2015	3	9	9	0	100
2016	13	39	37	2	95
2017	13	39	37	2	95
2018	8	24	24	0	100
2019	17	51	49	2	96

**Table 2:** Sample qualitative evaluation by the level of contamination in PT from 2014 to 2019.

Number of Larvae per Sample	Year/Number of Samples											
	2014/14		2015/3		2016/13		2017/13		2018/8		2019/17	
	C	I	C	I	C	I	C	I	C	I	C	I
0	14	0	3	0	13	0	13	0	8	0	17	0
1	10	4	3	0	8	5	11	2	7	1	17	0
3	14	0	3	0	11	2	12	1	8	0	17	0
5	14	0	3	0	13	0	12	1	8	0	15	2

C: correct; I: incorrect; 1—level not taken into account in the laboratories evaluation.

**Table 3:** Laboratory qualitative evaluation.

Year	Number of Laboratories Participating in the Study	Total Number of Laboratories with Correct Results	Total Number of Laboratories Reporting Incorrect Results	% of Laboratories That Passed PT Comparisons
2014	14	14	0	100
2015	3	3	0	100
2016	13	11	2	84.6
2017	13	12	1	92.3
2018	8	8	0	100
2019	17	15	2	88.2

**Table 4:** Descriptive statistics of reported results.

Parameters	Level 0	Level 1	Level 3	Level 5
Mean	0.00	0.82	2.35	3.63
Standard error	0.00	0.05	0.10	0.17
Median	0.00	1.00	3.00	4.00
Mode	0.00	1.00	3.00	5.00
Standard deviation	0.00	0.38	0.86	1.37
Sample variance	0.00	0.15	0.74	1.88
Kurtosis	Nd	1.04	0.62	0.22
Skewness	Nd	−1.74	−1.20	−0.84
Range	0.00	1.00	3.00	5.00
Minimum	0.00	0.00	0.00	0.00
Maximum	0.00	1.00	3.00	5.00
Sum of detected larvae	0.00	56.00	160.00	247.00
number	68.00	68.00	68.00	68.00
Confidence level (95%)	0.00	0.09	0.21	0.33

**Table 5:** Quantitative assessment.

Year	Number of Samples per Level		Number of Samples in Quantitative Assessment (*n* (%))				
		Level 0	Level 3	Level 3	Level 5	Level 5	Level 5
		|Δ| = 0 Correct	|Δ| ≤ 2 Correct	|Δ| > 3 Incorrect	|Δ| ≤ 2 Correct	|Δ| = 3 Doubtful *	|Δ| > 3 Incorrect
2014	14	14 (100)	14 (100)	0	11 (78.6)	3 (21.4)	0
2015	3	3 (100)	3 (100)	0	3 (100)	0	0
2016	13	13 (100)	11 (84.6)	2 (15.4)	11 (84.6)	2 (15.4)	0
2017	13	13 (100)	12 (92.3)	1 (7.7)	12 (92.3)	0	1 (7.7)
2018	8	8 (100)	8 (100)	0	6 (75)	2 (25)	0
2019	17	17 (100)	17 (100)	0	14 (82.3)	2 (11.7)	3 (17.6)

* Doubtful is qualified as positive laboratory assessment.

**Table 6:** Laboratory quantitative evaluation.

Year	Number of Laboratories Participating in the Study	Number Negatively Evaluated Laboratories	Number Positively Evaluated Laboratories
2014	14	0	14
2015	3	0	3
2016	13	2	11
2017	13	1	12
2018	8	0	8
2019	17	2	15

**Table 7:** Assessment of parameters characterizing the PT results.

Reference Value	Level 0	Level 1	Level 3	Level 5
s	0.00	0.38	0.86	1.37
X mean	0.00	0.82	2.35	3.63
CV	nd	0.46	0.36	0.37

s—standard deviation; CV—coefficient of variation.

**Table 8:** The PT’s results used for the calculation of sensitivity, specificity, and accuracy of the MSD for horse meat matrix.

Contamination Level	Species of *Trichinella* ssp.	Number of Examined Samples	Number of Samples Positively Assessed	Number of Samples Negatively Assessed
Negative: 0	*T. spiralis*	68	68	0
Low level: 3	*T. spiralis*	68	65	3
High level: 5	*T. spiralis*	68	65	3

**Table 9:** Basic parameters describing the MSD for horse meat as a matrix.

Validation Parameters (%)	Value	Upper Confidence Interval (UCL)	Lower Confidence Interval (LCL)
Specificity	100	100	100
Sensitivity	95.6	93.8	97.3
Accuracy	97.1	95.9	98.2

**Table 10:** Comparison of descriptive statistic parameters for horse and pork meat analysis.

Sample Matrix	Horse Meat		Pork Meat	
Number of Larvae Added to Samples	3	5	3	5
Mean	2.35	3.63	2.14	3.51
Standard error	0.10	0.17	0.02	0.03
Median	3.00	4.00	2.00	4.00
Mode	3.00	5.00	3.00	5.00
Standard deviation	0.86	1.37	1.01	1.39
Sample variance	0.74	1.88	1.02	1.94
Kurtosis	0.62	0.22	0.87	−0.22
Skewness	−1.20	−0.84	−0.39	−0.66
Range	3.00	5.00	9.00	7.00
Minimum	0.00	0.00	0.00	0.00
Maximum	3.00	5.00	9.00	7.00
Sum	68.00	68.00	4057.00	6646.00
Confidence level (95.0%)	0.21	0.33	0.05	0.06

**Table 11:** Comparison of parameters characterizing the MSD for horse and pig meat.

Validation Parameters	Horse Meat	Pork Meat
Specificity (%)	100	97.3
Sensitivity (%)	95.6	86.5
Accuracy (%)	97.1	89.2
LOD	1.14	1.08
LOQ	3.42	3.08

## Data Availability

Not applicable.
